# In Vitro Evaluation of Oxoplatin: An Oral Platinum(IV) Anticancer Agent

**DOI:** 10.1155/2009/348916

**Published:** 2009-06-30

**Authors:** Ulrike Olszewski, Florian Ach, Ernst Ulsperger, Gerhard Baumgartner, Robert Zeillinger, Patrick Bednarski, Gerhard Hamilton

**Affiliations:** ^1^Ludwig Boltzmann Cluster Translational Oncology, Operngasse 6/5, 1010 Vienna, Austria; ^2^Medical University of Vienna, Department of Gynecology, Waehringer Guertel 18-20, 1090 Vienna, Austria; ^3^Institute of Pharmacy, University of Greifswald, Friedrich-Ludwig-Jahn-Strasse 17, 17487 Greifswald, Germany

## Abstract

Platinum(IV) compounds like oxoplatin (*cis, cis, trans*-diammine-dichlorido-dihydroxido-platinum(IV)) show increased stability and therefore can be applied orally. In a panel of 38 human cancer cell lines this drug induced S-phase arrest and cell death with IC_50_ values 2.5-fold higher than cisplatin. Oxoplatin may be converted to cisplatin by intracellular reducing agents, however, exposure to 0.1 M HCl mimicking gastric acid yielded cis-diammine-tetrachlorido-platinum(IV) exhibiting twofold increased activity. Similar results were obtained for another platinum(IV) compound, JM 149 (ammine-dichlorido-(cyclohexylamine)-dihydroxido-platinum(IV)), but not for its parent drug JM 216/satraplatin. Genome-wide expression profiling of H526 small cell lung cancer cells treated with these platinum species revealed clear differences in the expression pattern of affected genes between oxoplatin and cisplatin. In conclusion, oxoplatin constitutes a potent oral agent that is either reduced or converted to distinct active compounds, for example, by gastric acid or acidic areas prevailing in solid tumors, in dependence of the respective pharmaceutical formulation.

## 1. Introduction

Cisplatin (*cis*-diammine-dichlorido-platinum(II)) was established as drug against diverse tumor types including testicular, ovarian, head and neck, bladder, esophageal, and small cell lung cancer (SCLC) [1, 2]. However, cisplatin exhibits only limited activity against tumors like colon and breast cancer and causes considerable side effects, and induction of resistance occurs frequently [3]. An auspicious “second generation” of platinum(II)-based drugs introduced into the clinic included carboplatin with similar cytotoxicity but less side effects than cisplatin, and oxaliplatin with antiproliferative effects even in cancers insensitive to cisplatin, for example, advanced colorectal tumors [4].

Platinum(II) drugs are known to act by direct binding to deoxyguanosine and deoxyadenosine molecules of DNA with formation of intrastrand crosslinks mainly and, to a minor degree, mono- and interstrand adducts [5]. In the recent years, several platinum analogs were developed, for example, diamminecyclohexyl- or ethylenediammine-analogs, nedaplatin, lobaplatin, ormaplatin, iproplatin as well as photoactivable and polynuclear complexes, respectively [6–11].

Molecules containing a platinum(IV) central atom provide several advantages over platinum(II) drugs, such as increased kinetic inertness and reduced reactivity, resulting in prolonged stability in the bloodstream, lower toxicity, and efficacy in cisplatin-resistant tumor cell lines [12, 13]. Due to their increased stability platinum(IV) complexes may be furthermore suitable for oral application. Distinct pharmacokinetic characteristics of new cytotoxic platinum(IV) complexes are determined by different axial ligands [14]. For example, satraplatin (bis-acetato-ammine-dichloro-cyclohexylamine-platinum(IV); JM 216), an orally active analog of carboplatin, was one of the first “third-generation” agents that showed evidence of antitumor activity [15]. In a phase III trial that investigated satraplatin in pretreated hormone-refractory prostate cancer patients the drug improved end points, such as progression-free survival, prostate-specific antigen response, and pain response, however, the difference in overall survival did not reach statistical significance [16]. 

New platinum(IV) compounds are under preclinical development and show promising activity in vitro, including trimethylplatinum(IV) complexes with cytosine ligands, metallacrown ethers, and O,O′-di-2-alkyl-(S,S)-ethylenediamine-N,N′-di-2-propanoate or ethylenediamine-N,N′-diacetate diester ligands of tetrachlorido-platinum(IV) complexes [17–20].

Since it is generally accepted that reduction of platinum(IV) has to occur prior to binding to the target DNA, platinum(IV) molecules are believed to represent relatively stable prodrugs that are able to compass the tumor tissue largely without being reduced in the bloodstream, enter the cell, and reach their target intact [21–24]. Only following reduction the resulting platinum(II) species bind to DNA and inhibit replication and transcription by formation of intra- and/or interstrand adducts resulting in cell cycle arrest in the G2M phase and programmed cell death [13, 16]. Cellular reducing agents like ascorbic acid, thiol-containing species like metallothioneins and glutathione, and others may trigger an activation of platinum(IV) prodrugs [25]. 

A further orally applicable platinum(IV) anticancer drug currently under development is *cis, trans, cis*-diammine-dihydroxido-dichlorido-platinum(IV) (oxoplatin) which was synthesized by Chugaev and Khlopin for the first time in the Russian Federation in 1927 [26]. Not until 1977, its cytotoxic activity was demonstrated in rat tumor models [27]. Presnov et al. compared antitumoral and pharmacokinetic properties of oxoplatin with that of cisplatin: therapeutic and maximal tolerated doses (MTDs) were tenfold higher for oxoplatin than for cisplatin. Furthermore, oxoplatin exhibited a prolonged therapeutic effect, antimetastatic activity, and similar or even higher tumor growth inhibition following eight days of either intraperitoneal, intramuscular, subcutaneous, oral, or rectal treatment. The MTD of perorally dispensed oxoplatin was two times lower in comparison to all other administration routes, and no complete cross-resistance was found for cisplatin and oxoplatin. The absence of necrotic lesions of the kidneys that are commonly observed for cisplatin pointed to a much lower nephrotoxicity of oxoplatin. Finally, oxoplatin left the blood-flow much more rapidly than cisplatin and showed significantly shorter half-elimination from blood than cisplatin. However, one study reported significant necrosis in the distal tubules of kidneys of rats triggered by oxoplatin [28].

Native platinum(IV) compounds can bind to DNA directly, however, this process is such slow, that it is supposed to be of no clinical relevance. Therefore in the present paper the in vitro cytotoxicity of oxoplatin and its possible activation by reduction through exposure to hydrochloric and ascorbic acid were investigated. The structure and pharmacokinetic characteristics of an orally applied drug may be chemically modified during gastric passage. If the compound is not inert, the result may be either activation or inactivation by the prevailing acidic conditions in the stomach. Since oxoplatin may represent a prodrug of cisplatin, the intracellular effects of both platinum drugs on gene expression patterns of a sensitive cell line were compared using microarrays for genome-wide expression analysis.

## 2. Materials and Methods

### 2.1. Chemical Reagents

**Figure 1:**
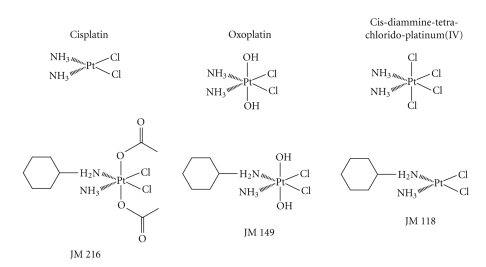
Chemical structures of the platinum compounds used in the present study.

Unless otherwise noted, all chemicals were obtained from Sigma-Aldrich (St. Louis, Mo, USA). Dulbecco's phosphate buffered saline (PBS) was purchased from Gibco/Invitrogen (Carlsbad, Calif, USA). Oxoplatin (*cis, cis, trans*-diammine-dichlorido-dihydroxido-platinum(IV)), trans-oxoplatin (trans, trans, trans-diammine-dichlorido-dihydroxido-platinum(IV)), *cis*-diammine-tetrachlorido-platinum(IV), satraplatin (JM 216; bis-acetato-ammine-dichlorido-cyclohexylamine-platinum(IV)), JM 149 (ammine-dichlorido-(cyclohexylamine)-dihydroxido-platinum(IV)), and JM 118 (ammine-dichlorido-(cyclohexylamine)-platinum(II)) were synthesized according to standard procedures by Chiracon, Luckenwalde, Germany, and kindly provided by IPSS, Berlin, Germany (Figure 1). All compounds were prepared as stock solutions of 2 mg/mL in either DMSO (cisplatin, *cis*-diamminetetrachloroplatinum(IV)) or in 0.9% NaCl solution sterilized by filtration, and aliquots stored at −20°C.

### 2.2. Derivatization of Oxoplatin

Solutions containing oxoplatin at a concentration of 120 *μ*M were acidified to achieve a final concentration of 0.1 M HCl, incubated at room temperature for 15 minutes and finally pH was adjusted to pH = 7.4 with NaOH for their use in proliferation assays. The putative derivate *cis*-diammine-tetrachloridoplatinum(IV) was synthesized and served as reference substance for infrared (IR) spectral analysis of the species resulting from addition of 1.1 g oxoplatin to 100 mL 0.1 M HCl, (37°C, 120 minutes) as well as for the in vitro experiments. The recorded IR spectra of *cis*-diammine-tetrachloridoplatinum(IV) and of the oxoplatin-HCl preparation proved to be undistinguishable (IR (*λ*[cm^−1^]): 3539 (w); 3334 (m); 3251 (s); 3174 (vs); 3075 (m); 2663 (w); 2624 (w); 2397 (w); 2189 (w); 1675 (m); 1556 (s); 1318 (s); 1309 (s); 1015 (w); 863 (w); 832 (w)) indicating conversion of the oxoplatin to *cis*-diammine-tetrachloridoplatinum(IV). For reduction of oxoplatin the compound was treated with ascorbic acid (5 mM, 24 hours, 37°C) prior to application.

### 2.3. Cell Lines and Culture Conditions

Cell lines were obtained from American Type Culture Collection (ATCC, Rockville, Md, USA), with the exception of melanoma cell lines M518, M607, and JVSO (Department of Dermatology, Medical University of Vienna) and the Ewing's sarcoma/PNET (peripheral neuroectodermal tumor) cell lines (our institution and cooperations). Cells were grown RPMI-1640 bicarbonate medium (Seromed, Berlin, Germany) supplemented with 10% fetal bovine serum (Seromed) and 4 mM glutamine in a humidified incubator (Heraeus Cytoperm, Hanau, Germany; 5% CO_2_, 37°C, 95% humidity). Cells were checked for mycoplasma contamination (Mycoplasma PCR ELISA, Roche Diagnostics, Vienna, Austria). Attached cells were subcultured by trypsination (0.05% trypsin containing 0.02% EDTA) two times a week. Cell numbers were counted using a microcellcounter (CC110, Sysmex TOA, Tokyo, Japan).

### 2.4. MTT Chemosensitivity Assay

1 × 10^4^ cells in 100 *μ*L medium/well were distributed to 96 well microtiter plates (Greiner, Kremsmuenster, Austria), and substances to be tested were added in a volume of another 100 *μ*L. All compounds were serially diluted in 6–10 twofold steps in triplicate. The microtiter plates were incubated under tissue culture conditions (RPMI-1640/10% FCS, 4 mM glutamine; 37°C, 5% CO_2_, 95% humidity) for four days, and cell viability was measured using a modified MTT (3-(4,5-dimethylthiazol-2-yl)-2,5-diphenyl-tetrazolium bromide) assay (EZ4U, Biomedica, Vienna, Austria). This assay quantifies mitochondrial activity and thereby cell viability by generation of formazane dye (optical density at 450 nm) from tetrazolium salts by mitochondrial reduction. Optical density was measured in a microplate reader (Eurogenetics, Brussels, Belgium) at 450 nm with an empty well as reference. Test results for slowly and rapidly proliferating cells were recorded between optical densities of 0.3 and 1.5, respectively. Wells containing media alone were used as controls and values obtained set to 100% proliferation.

### 2.5. Cell Cycle Analysis

1 × 10^6^ cells were cultivated in 6-well plates and incubated with the compound of interest for four days. Thereafter, cells were trypsinized, washed in PBS, fixed in 70% ethanol at −20°C for 30 minutes, washed again, transferred into staining solution (20 *μ*g/mL propidium iodide (PI), 5 *μ*g/mL ribonuclease (RNase) A and 0.05% Nonidet P40 in PBS), and incubated at room temperature (RT) overnight. Washed cells were analyzed by flow cytometry (Cytomics FC500, Beckman Coulter, Krefeld, Germany) at excitation and emission wavelengths of 488 and 675 nm, respectively, acquiring 1 × 10^4^ cells per run. MultiCycle AV software (Phoenix Flow Systems, San Diego, Calif, USA) was employed for calculation of cell cycle distribution from linear PI histograms. Percentages of cells in cell cycle phases G1/0 (resting), S (DNA synthesis), and G2M (mitotic cells) were recorded. Experiments were done in duplicate.

### 2.6. Flow Cytometric Measurement of Reactive Oxygen Species

Intracellular generation of reactive oxygen species (ROS) induced by platinum compounds was measured by flow cytometry using dihydroethidium (DHE) for detection of superoxide anion production. Treated and untreated cells (0.5 × 10^6^) in 6-well plates were incubated in RPMI-1640 medium containing 5 *μ*M DHE at 37°C for 15 minutes. Dye oxidation was quantified using flow cytometry with excitation and emission settings of 488 and 610 nm, respectively.

### 2.7. Gene Expression Analysis

For gene expression analysis, approximately 30 × 10^6^ H526 SCLC cells were treated with 4.1 *μ*M cisplatin or 3.7 *μ*M oxoplatin, respectively, in tissue culture flasks for three days (37°C, 5% CO_2_, 95% humidity). In brief, cells were harvested by trypsination and pellets of approximately 30 × 10^6^ cells stored frozen at –80°C before they were extracted with 2.55 mL extraction buffer (4 M guanidine isothiocyanate, 0.5% sodium N-lauroylsarcosinate, 10 mM EDTA, 5 mM sodium citrate, 100 *μ*M *β*-mercaptoethanol) at 4°C. DNA and RNA of the lysates were separated by cesium trifluoroacetate ultracentrifugation. RNA was washed with ice cold 96% ethanol and dissolved in water in order to check for content and purity by measurements of optical density at 260/280 nm. Gene expression analysis was performed using the Applied Biosystems Human Genome Survey Microarray V2.0 (Applied Biosystems, Foster City, Calif, USA). Therefore, 2–5 *μ*g mRNA (20–50 *μ*g total RNA) were reversely transcribed to first-strand cDNA and labeled with digoxigenin-UTP according to the Applied Biosystems Chemiluminescent Reverse Transcription protocol. RNA was degraded and cDNA purified using DNA purification columns (Pico/Fresco—Heraeus, Hanau, Germany). Hybridization of the cDNA and microarray analysis was performed according to the Applied Biosystems Chemiluminescence Detection Kit protocol using a hybridization oven (Infors HT Minitron, Bottmingen, Switzerland) for incubation of the microarray cartridges and the Applied Biosystems 1700 Chemiluminescent Microarray Analyzer for evaluation. Results for oxoplatin- or cisplatin treatment were then evaluated in comparison to the gene expression pattern of H526 control cells using the Microsoft Excel software.

### 2.8. Statistics

Values are demonstrated as means ± SD. Statistical analysis was done using Student's *t*-test for samples with normal distribution (*p* < .05 is regarded as statistical significant) or Kruskal-Wallis test as nonparametric method.

## 3. Results

### 3.1. Screening of the Antiproliferative Activity of Oxoplatin in a Panel of Human Tumor Cell Lines

**Figure 2:**
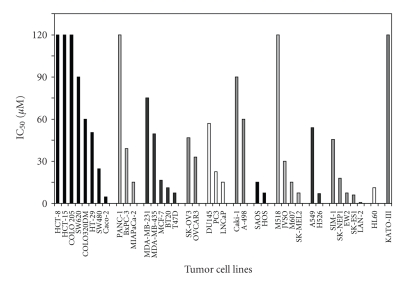
In vitro cytotoxicity of oxoplatin against a panel of human cancer cell lines. Mean IC_50_ values for oxoplatin were obtained from triplicate dose-response curves (sd < 8%). Cells were incubated under tissue culture conditions for four days and viable cells detected using a modified MTT assay.

To investigate the in vitro antiproliferative activity of oxoplatin a panel of 38 human cancer cell lines was initially screened by incubation of cells with the compound at concentrations of 0.6–240 *μ*M for four days. Oxoplatin exhibited partial activity throughout the tumor entities tested. IC_50_ values ranged between 0.6–120 *μ*M with a mean IC_50_ of 22.8 ± 17.4 *μ*M for sensitive cell lines defined by an IC_50_ below 60 *μ*M (Figure 2). The mean IC_50_ for cisplatin and the same sensitive cell lines was 10.1 ± 9.1 *μ*M, respectively. The ovarian cancer cell lines SK-OV3 and OVCAR3, and the renal cancer cell lines Caki-I and A-498 were most insensitive, whereas each of the remaining tumor entities included cell lines with considerable sensitivity to oxoplatin.

### 3.2. Effect of Pretreatment of the Platinum Compounds with Acids

#### 3.2.1. Activation of Oxoplatin by 0.1 M HCl

In the following experiments, dose-response curves were obtained for oxoplatin, cisplatin, JM 216, and JM 149, either applied in native form or following exposure to 0.1 M HCl at room temperature for 15 minutes, in proliferation assays using COLO 205 cells. Concentrations of the platinum compounds ranged from 0.6–120 *μ*M, and cells were incubated under tissue culture conditions for four days.

**Figure 3:**
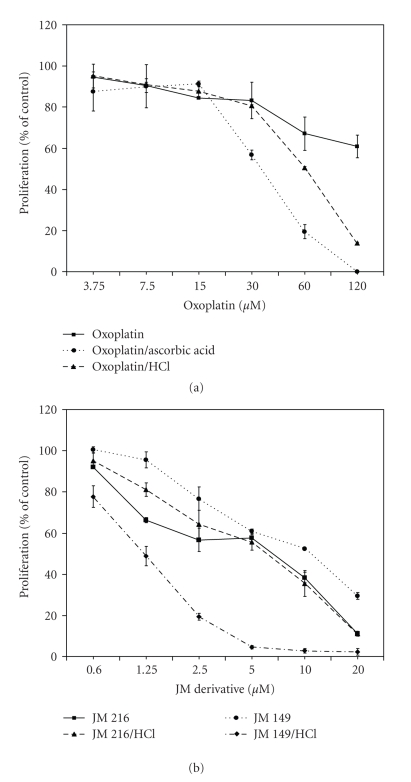
Dose-response curves obtained in COLO 205 cells of (a) native oxoplatin and oxoplatin after incubation with 0.1 M HCl for 15 minutes or 5 mM ascorbic acid for 24 hours, and of (b) JM 216 or JM 149, respectively, also either as native compound or after exposure to 0.1 M HCl for 15 minutes.

The antiproliferative activity of cisplatin was not affected by 0.1 M HCl, however, oxoplatin exhibited a twofold enhanced cytotoxicity after preincubation under highly acidic conditions. JM 216 was affected to a minor degree, whereas the activity of JM 149 was also markedly increased (>8-fold) by exposure to 0.1 M HCl. Figures 3(a)  and 3(b)  show dose-response curves of oxoplatin, JM 216, and JM 149, with or without HCl-pretreatment, respectively. The *trans*-acetato ligands of JM 216 protect against substitution by 0.1 M HCl resulting in a dose-response curve similar to the native drug. In contrast, the metabolite of JM 216 lacking the *trans*-acetato groups, JM 149, exhibited pronounced cytotoxic activity following activation by 0.1 M HCl similar to oxoplatin. The inactive stereoisomer *trans*-oxoplatin (all-*trans*-diammine-dichlorido-dihydroxidoplatinum(IV)) was not affected by treatment with 0.1 M HCl (data not shown). Similar results regarding increases of the cytotoxicity of oxoplatin by 0.1 M HCl were obtained in a panel of other cell lines (data not shown).

Exposure of 120 *μ*M oxoplatin to 6.3–100 *μ*M HCl prior to application to COLO 205 colon cancer and BxPC-3 pancreatic cancer cells revealed minimal concentrations of 12.5–25.0 *μ*M HCl to be required for activation of oxoplatin, within an incubation time of 10 minutes.

The significant enhancement of the cytotoxicity of oxoplatin by 0.1 M HCl strongly points to a chemical modification of the drug at low pH. Accordingly, comparison of the anticancer activities of HCl-treated oxoplatin with *cis*-diammine-tetrachloridoplatinum(IV) in chemosensitivity tests revealed no differences in cytotoxicity of the two preparations (data not shown).

#### 3.2.2. Activation of Oxoplatin by Physiological Concentrations of Ascorbic Acid

Similar to the experiments using HCl mentioned above, proliferation assays were carried out where the platinum compounds were added to physiological intracellular concentrations of ascorbic acid (5 mM) prior to application, and growth inhibition was compared to the untreated drug. Dose-response curves of 3.75–120 *μ*M oxoplatin following exposure to ascorbic acid revealed increased antiproliferative activity of oxoplatin in COLO 205 cells during four days of incubation (Figure 3(b)). The resulting cytotoxicity was comparable to that of cisplatin. In addition, pretreatment of oxoplatin with *β*-mercaptoethanol did not lead to an alteration of the antiproliferative effect of oxoplatin (data not shown).

### 3.3. Alterations in Cell Cycle Distribution Induced by Cisplatin and Oxoplatin in Colo 205 Cells

**Figure 4:**
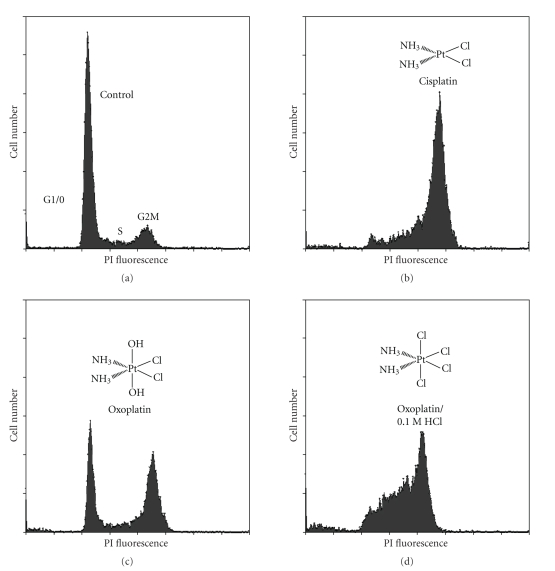
Comparison of cell cycle perturbations observed in COLO 205 colon cancer cells induced by the various platinum compounds tested here. Cells were grown in (a) medium alone, (b) medium containing 65 *μ*M cisplatin, (c) 120 *μ*M oxoplatin, and (d) 120 *μ*M oxoplatin following treatment with 0.1 M HCl, respectively, under tissue culture conditions for four days, and subsequently stained with propidium iodide (PI). The concentrations of the platinum compounds used had resulted in comparable cytotoxicity in preceding proliferation tests.

To analyze alterations of cell cycle distribution in COLO 205 colon cancer cells in response to distinct platinum compounds concentrations of the substances that had revealed similar cytotoxic activity in previous proliferation assays were used. Thus, the effects of 65 *μ*M cisplatin, 120 *μ*M oxoplatin, and 120 *μ*M oxoplatin treated with 0.1 M HCl, respectively, were determined using PI staining of DNA and flow cytometry (Figure 4). In comparison to cisplatin (accumulation of cells in S- and mainly G2M-phase), oxoplatin induced partial arrest of cells in G2M phase, while the residual cells remained in G1/0. Interestingly, exposure of oxoplatin to 0.1 M HCl prior to addition to the cells resulted in cell cycle distribution with pronounced accumulation of cells in S and G2/M phases very similar to that of cisplatin. The same cell cycle pattern was observed for *cis*-diammine-tetrachloridoplatinum(IV) (data not shown).

### 3.4. Generation of ROS Triggered by Platinum Compounds

**Figure 5:**
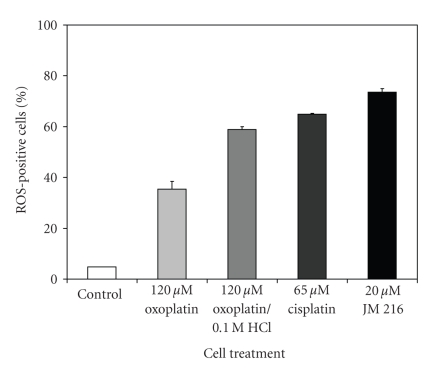
Platinum compound-induced generation of ROS in COLO 205 cells. Cells were treated with the respective platinum compound for four days and labeled with DHE for detection of superoxide production by flow cytometry. Data are represented as mean ± SD, *n* = 3.

Intracellular generation of ROS induced by platinum compounds was measured by analysis of cells labeled with dihydroethidium (DHE) by flow cytometry. A comparison of the potency of the induction of ROS-generation by concentrations of the substances yielding similar cytotoxicity revealed highest levels of ROS (Figure 5) by 20 *μ*M JM 216 (73.5 ± 1.5%), 65 *μ*M cisplatin (64.9 ± 0.3%), 0.1 M HCl-pre-exposed oxoplatin (58.9 ± 1.8%), and 120 *μ*M oxoplatin (35.5 ± 3.0%). These results are in good agreement with the increased cytotoxic activity of oxoplatin following treatment with 0.1 M HCl, however, the ROS-generating capacities of JM216 and cisplatin correlate poorly with their respective antitumor effects, possibly due to different intracellular effects and kinetics.

### 3.5. Comparative Microarray Gene Expression Analysis

**Figure 6:**
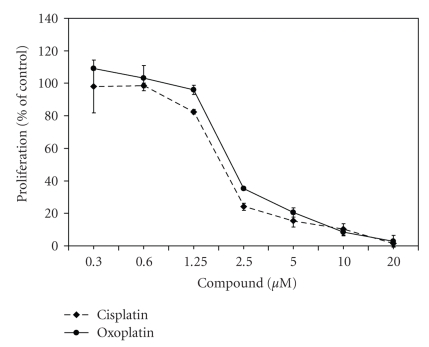
Dose-response curves of cisplatin and oxoplatin obtained in H526 SCLC cells (mean ± SD, *n* = 3). H526 cells were chosen for comparative microarray gene expression analysis, since the two platinum compounds exhibited similar cytotoxicity, especially in this cell line.

As shown in Figure 6, cisplatin and oxoplatin exhibited similar antiproliferative effects in the chemosensitive SCLC cell line H526. This cell line is characterized by more rapid and pronounced acidification of the culture medium (pH < 6.8) than other cell lines. These comparable IC_50_ concentrations of cisplatin and oxoplatin point to an activation of oxoplatin by chemical modification involving the extracellular/intracellular acidic conditions. Therefore H526 cells were chosen to compare cisplatin- or oxoplatin-induced alterations in genome-wide gene expression in cultures treated with concentrations of the respective compound that resulted in sublethal toxicity (>95% viability).

Of the 55 genes upregulated more than fourfold in oxoplatin-treated H526 cells only three genes, namely, HIST2H2BE, PNUTL2, and AKR1C3, were in common with those genes upregulated in cisplatin-treated cells (the 27 genes exhibiting the strongest increases in expression triggered by oxoplatin are shown in Table 1). In regard to contrary gene expression, 2/55 genes were upregulated in oxoplatin-treated and downregulated in cisplatin-treated cells (RASD2, RASD family member 2; SAT, spermine N1-acetyltransferase) and 0/150 genes under the reverse conditions of up- and downregulation. Much more genes (73/302) were downregulated both in oxoplatin-treated and cisplatin-treated H526 cells (the 37 genes exhibiting the strongest reduction in expression triggered by oxo- or cisplatin are shown in Table 2). Cytotoxicity of oxoplatin was evidenced by DNA damage-inducible genes, generation of ROS by dual oxidase 2 (DUOX2), and cell death was most likely linked to increased expression of caspase 3 (Table 1). Downregulated genes common for oxoplatin and cisplatin include those coding for antiapoptotic proteins (TNFRSF6B and BCL2L1) as well as a number of genes associated with regulation of the cytoskeleton and cellular metabolism (Table 2). Although oxoplatin and cisplatin showed a partial overlap of the expression of affected genes in H526 cells, almost 80% of these genes were different for the two drugs. This finding strongly points to different mechanisms of action of cisplatin and oxoplatin that are not consistent with the role of oxoplatin and derived species as inactive prodrugs of an exclusively active cisplatin.

## 4. Discussion

Cisplatin has proved to be a potent cytotoxic agent in the therapy of cancer [2]. In the last years, great importance has been placed on the development of orally applicable anticancer drugs in order to allow outpatient care. Among the first oral platinum coordination complexes established are picoplatin and satraplatin that have shown promise in preclinical and clinical trials [4]. Oral picoplatin is currently being checked in a Phase-I study in patients with solid tumors. Satraplatin (JM 216) showed promise in patients with hormone-refractory prostate cancer and was considered for approval by the Food and Drug Administration in 2007. However, the satraplatin SPARC phase III trial in second-line hormone refractory prostate cancer failed to improve overall survival [16].

Oxoplatin represents another platinum(IV) compound that may be administered orally. Pharmacokinetic studies using ^191^Pt-labeled oxoplatin in melanoma B-16—bearing mice showed rapid distribution throughout the blood and most organs with a decline of the blood level after 1 hour of intravenous drug administration. Elimination from the blood followed a biexponential course with higher half-lives in melanoma B-16—bearing mice than in controls [29, 30]. The highest accumulation of oxoplatin was found in kidneys, liver, spleen, adrenals, thymus, skin, and the tumor, respectively. Since oxoplatin was rapidly excreted by the kidneys it was regarded to possess only low nephrotoxicity. Comparison of the pharmacokinetics of oxoplatin and cisplatin revealed higher plasma levels of the former following injection, and oxoplatin exhibited lower protein binding. Oxoplatin was furthermore found to accumulate in tumor tissue, and metabolization resulted in the formation of several molecules, amongst them cisplatin pointing to the role of oxoplatin being a prodrug of cisplatin, however, this hypothesis has not been proved so far. 

As shown in the present paper, oxoplatin exhibits cytotoxicity in a broader spectrum of tumor entities in vitro, such as cancers of the colon, pancreas, breast, prostate as well as melanoma, tumors of childhood, and osteosarcoma with IC_50_ levels ranging from 0.6–120 *μ*M. Still, the mechanism of action of platinum coordination complexes is not fully understood. It is generally believed that platinum(IV) compounds need to be reduced in order to bind to the DNA, however, Novakova and others demonstrated that platinum(IV) complexes can bind to DNA directly without previous reduction, even though more slowly and to a lesser extent than cisplatin [21–24, 31–34]. Cisplatin-DNA adducts lead to a conformational change of the DNA double-helix allowing protein binding of molecules containing high mobility group domains impairing DNA replication and transcription [35–38]. Our results demonstrate that oxoplatin induced cell cycle arrest in S and G2M phases, generation of ROS, and cell death. In contrast, cisplatin led to accumulation of cells mostly in G2M phase, indicating differences in the modes of action of the two platinum compounds. Furthermore, exposure of oxoplatin to 0.1 M HCl mimicking the low pH of gastric acid prior to application to the cells yielded cell cycle arrest mainly in S-phase. In comparison to that, the satraplatin metabolites JM 149 and JM 118, representing cyclohexyl-substituted cis- or oxoplatin analogs, triggered arrest mainly in S and G2M or G2M phases, respectively, similar to cisplatin or oxoplatin (data not shown). 

Reduction of platinum(IV) compounds may take place in the bloodstream or intracellularly and is accomplished by reducing agents such as ascorbic acid, methionine, cysteine, glutathione, uric or lactic acid, and sulfhydryl-group containing proteins. For example, human serum albumin, the predominant plasma protein mediating transport of drugs and other (macro)molecules in the bloodstream, features reactive thiol groups of cysteine as well as methionine residues, which may also constitute a binding partner for platinum compounds [39, 40]. In contrast to a platinum(II) analog, binding of platinum(IV) compounds to serum albumin in vitro was reported to be very low and dependent on the respective reduction potentials of the molecules [12]. The axial ligands bound to the platinum(IV) center determine reduction potential, stability under reducing conditions as well as reactivity towards DNA-binding [12, 41]. These properties are conferred by the leaving groups and decrease following the order chlorido > acetato > hydroxido, implying that chlorido-substituents facilitate the conversion of platinum(IV) into platinum(II) [42]. The effects of reducing agents on the activity of oxoplatin were assessed in comparison to cisplatin in the present paper.

With respect to an oral drug, the normally very low pH prevailing in the stomach that may be responsible for a chemical modification of the compound must be considered. As a cell line relatively insensitive to oxoplatin but exhibiting moderate sensitivity to cisplatin, COLO 205 colon cancer cells were chosen to investigate a possible relevance of highly acidic conditions on the potency of oxoplatin or the other platinum(IV) compounds. Treatment of oxoplatin with 0.1 M HCl led to an approximately twofold increase in the cytotoxicity that was comparable to that of cisplatin. Cisplatin did not exhibit altered activity following treatment with HCl. Administered orally, JM 216 is metabolized into JM 149 and JM 118 exhibiting cytotoxic effects for themselves following the order JM 118 > JM 216 > JM 149 [16]. JM 118 was inert to 0.1 M HCl, as was JM 216, however, JM 149, the compound analogous to oxoplatin, revealed a considerably increased cytotoxicity. Therefore the fate and activity of the oral prodrug oxoplatin critically depend on its formulation, that can be chosen to allow for release in the stomach or part of the intestine. To figure out whether oxoplatin was actually transformed into the putative derivate *cis*-diammine-tetrachlorido-platinum(IV), this molecule was synthesized and compared to 0.1 M HCl-treated oxoplatin. *Cis*-diammine-tetrachlorido-platinum(IV) yielded the same cytotoxic effect as HCl-treated oxoplatin and was proved to be identical in IR spectroscopy. Due to the high reduction potential of the chloride-ligands, it may be hypothesized that those may be easily removed and the molecule converted into platinum(II), however, as will be explained below, some further findings point to different mechanisms of action of cisplatin and oxoplatin.

Cisplatin and oxoplatin exhibited similar antiproliferative effects in the H526 cell line that was found to acidify the extracellular medium to a significant degree (pH < 6.8). Therefore, chemosensitive H526 cells were selected for genome-wide microarray analysis of cisplatin- or oxoplatin-induced gene expression. According to the ATCC, the H526 cell line, initiated using a bone metastasis of SCLC prior to therapy, shows expression of neuron-specific enolase, brain enzyme of creatine kinase, and p53 mRNA. This line produces colonies in soft agar and tumors in athymic nude mice. Comparison of the gene expression patterns of controls and treated cells revealed significant differences in the expression pattern of target genes for the two platinum compounds, with only three upregulated genes in common. Approximately one fourth of the downregulated genes are shared between oxoplatin- and cisplatin-treated H526 cells pointing to common pathways in the suppression of genes involved in platinum-induced cell death. Therefore the mode of action of oxoplatin involves a majority of unique affected genes, that are clearly different from those altered by cisplatin.

## 5. Conclusion

In summary, oxoplatin exhibits in vitro activity against diverse human tumor cells, inducing growth arrest, generation of ROS and cell death. Depending on its formulation this drug is converted to * cis*-diammine-tetrachlorido-platinum(IV) under acidic conditions or reduced in the presence of ascorbic acid. Comparative genome-wide gene expression analysis using a chemosensitive SCLC cell line demonstrates that the majority of the oxoplatin- and cisplatin-regulated target genes are not identical and therefore oxoplatin represents not a simple prodrug of cisplatin.

## Figures and Tables

**Table 1:** Selected genes exhibiting > fourfold upregulated expression in H526 cells that were treated with oxoplatin assorted according to their cellular function. H526 SCLC cells were either left untreated or treated with 3.75 *μ*M oxoplatin, respectively, in tissue culture flasks for three days. Thereafter, RNA of control and treated cells was extracted, and gene expression was analyzed using the Applied Biosystems Human Genome Survey Microarray V2.0.

Cellular process	Locus link	Gene symbol	Gene name
Transcription	2306	FOXD2	Forkhead box D2
	5450	POU2AF1	POU domain, class 2, associating factor 1
	1649	DDIT3	DNA-damage-inducible transcript 3

Apoptosis	84301	DDI2	DNA-damage inducible protein 2
	9518	GDF15	Growth differentiation factor 15
	836	CASP3	Caspase 3, apoptosis-related cysteine protease
	8349	HIST2H2BE	Histone 2, H2be

Cytoskeleton	649	BMP1	Bone morphogenetic protein 1
	2620	GAS2	Growth arrest-specific 2
	5414	PNUTL2	Peanut-like 2 (Drosophila)
	5100	PCDH8	Protocadherin 8

Signal transduction	10673	TNFSF13B	Tumor necrosis factor (ligand) superfamily, member 13b
	10368	CACNG3	Calcium channel, voltage-dependent, gamma subunit 3
	8416	ANXA9	Annexin A9
	1184	CLCN5	Chloride channel 5 (nephrolithiasis 2, X-linked, Dent disease)
	147798	TMC4	Transmembrane channel-like 4

Metabolism	50506	DUOX2	Dual oxidase 2
	81894	SLC25A28	Solute carrier family 25, member 28
	3067	HDC	Histidine decarboxylase
	8644	AKR1C3	Aldo-keto reductase family 1, member C3
	6569	SLC34A1	Solute carrier family 34 (sodium phosphate), member 1
	10404	PGCP	Plasma glutamate carboxypeptidase
	9709	HERPUD1	Homocysteine-inducible, endoplasmic reticulum stress-inducible, ubiquitin-like domain member 1
	6303	SAT	Spermidine/spermine N1-acetyltransferase

Transport	1520	CTSS	Cathepsin S
	27074	LAMP3	Lysosomal-associated membrane protein 3
	4189	DNAJB9	DnaJ (Hsp40) homolog, subfamily B, member 9

**Table 2:** Selected genes exhibiting > fourfold commonly downregulated expression in H526 cells treated with oxoplatin or cisplatin compared to untreated control cells classified according to their cellular function. For gene expression analysis H526 SCLC cells were treated with 3.75 *μ*M oxoplatin or 4.1 *μ*M cisplatin, respectively, in tissue culture flasks for three days. Thereafter, RNA of control and treated cells was extracted, and gene expression was analyzed using Applied Biosystems Human Genome Survey Microarray V2.0.

Cellular process	Locus link	Gene symbol	Gene name
Transcription	4150	MAZ	MYC-associated zinc finger protein (purine-binding transcription factor)
	3221	HOXC4	Homeo box C4
	10658	CUGBP1	CUG triplet repeat, RNA binding protein 1

Apoptosis	8771	TNFRSF6B	Tumor necrosis factor receptor superfamily, member 6b, decoy
	598	BCL2L1	BCL2-like 1
	3855	KRT7	Keratin 7

Cytoskeleton	347733	MGC8685	Tubulin, beta polypeptide paralog
	823	CAPN1	Calpain 1, (mu/I) large subunit
	50861	STMN3	Stathmin-like 3
	5962	RDX	Radixin
	977	CD151	CD151 antigen
	50512	PODLX2	Endoglycan
	55920	TD-60	RCC1-like
	91179	SCARF2	Scavenger receptor class F, member 2
	6711	SPTBN1	Spectrin, beta, nonerythrocytic 1
	6596	SMARCA3	SWI/SNF related, matrix associated, actin dependent regulator of chromatin, subfamily a, member 3
	4134	MAP4	Microtubule-associated protein 4

Signal transduction	51582	OAZIN	Ornithine decarboxylase antizyme inhibitor
	26469	PTPN18	Protein tyrosine phosphatase, nonreceptor type 18 (brain-derived)
	11247	NXPH4	Neurexophilin 4
	53944	CSNK1G1	Casein kinase 1, gamma 1
	1445	CSK	C-src tyrosine kinase
	30851	TIP-1	Tax interaction protein 1
	23187	PHLDB1	Pleckstrin homology-like domain, family B, member 1
	3597	IL13RA1	Interleukin 13 receptor, alpha 1

Metabolism	2539	G6PD	Glucose-6-phosphate dehydrogenase
	5211	PFKL	Phosphofructokinase, liver
	2542	SLC37A4	Solute carrier family 37 (glycerol-6-phosphate transporter), member 4
	478	ATP1A3	ATPase, Na+/K+ transporting, alpha 3 polypeptide
	55611	OTUB1	OTU domain, ubiquitin aldehyde binding 1
	2030	SLC29A1	Solute carrier family 29 (nucleoside transporters), member 1
	6566	SLC16A1	solute carrier family 16 (monocarboxylic acid transporters), member 1

Transport	29924	EPN1	Epsin 1
	6844	VAMP2	Vesicle-associated membrane protein 2 (synaptobrevin 2)
	10527	IPO7	Importin 7
	7514	XPO1	Exportin 1 (CRM1 homolog, yeast)
	1654	DDX3X	DEAD (Asp-Glu-Ala-Asp) box polypeptide 3, X-linked
